# Environmental contaminants modulate the transcriptional activity of polar bear (*Ursus maritimus*) and human peroxisome proliferator-activated receptor alpha (PPARA)

**DOI:** 10.1038/s41598-019-43337-w

**Published:** 2019-05-06

**Authors:** Heli Routti, Mari K. Berg, Roger Lille-Langøy, Lene Øygarden, Mikael Harju, Rune Dietz, Christian Sonne, Anders Goksøyr

**Affiliations:** 1 Norwegian Polar Institute, Fram Centre, NO-9296 Tromsø, Norway; 2 Department of Biological Sciences, University of Bergen, NO-5020 Bergen, Norway; 3 Norwegian Institute for Air Research, Fram Centre, NO-9296 Tromsø, Norway; 4 Aarhus University, Department of Bioscience, Arctic Research Centre, DK-4000 Roskilde, Denmark

**Keywords:** Fat metabolism, Environmental impact, Mechanism of action

## Abstract

Peroxisome proliferator-activated receptor alfa (PPARA/NR1C1) is a ligand activated nuclear receptor that is a key regulator of lipid metabolism in tissues with high fatty acid catabolism such as the liver. Here, we cloned PPARA from polar bear liver tissue and studied *in vitro* transactivation of polar bear and human PPARA by environmental contaminants using a luciferase reporter assay. Six hinge and ligand-binding domain amino acids have been substituted in polar bear PPARA compared to human PPARA. Perfluorocarboxylic acids (PFCA) and perfluorosulfonic acids induced the transcriptional activity of both human and polar bear PPARA. The most abundant PFCA in polar bear tissue, perfluorononanoate, increased polar bear PPARA-mediated luciferase activity to a level comparable to that of the potent PPARA agonist WY-14643 (~8-fold, 25 μM). Several brominated flame retardants were weak agonists of human and polar bear PPARA. While single exposures to polychlorinated biphenyls did not, or only slightly, increase the transcriptional activity of PPARA, a technical mixture of PCBs (Aroclor 1254) strongly induced the transcriptional activity of human (~8-fold) and polar bear PPARA (~22-fold). Polar bear PPARA was both quantitatively and qualitatively more susceptible than human PPARA to transactivation by less lipophilic compounds.

## Introduction

Arctic wildlife populations are exposed to numerous environmental contaminants. In particular, species at the top of Arctic marine food webs, such as the polar bear (*Ursus maritimus*), accumulate high levels of organohalogen contaminants (OHCs) including polychlorinated biphenyls (PCBs), organochlorine pesticides, and perfluoroalkyl substances (PFASs)^1,2^. Numerous metabolites of OHCs, such as hydroxyl (OH) PCBs and MeSO_2_-PCBs/DDE, are also found in polar bear tissues, as the species is capable of efficient biotransformation of xenobiotic compounds^3,4^. Contaminant exposure in polar bears has been related to multiple health effects, such as endocrine and metabolic disruption, immune deficiency, and pathological disorders^5–9^. A recent correlative field study using biomarkers from transcriptome to metabolome indicates that contaminants also target polar bear lipid metabolism^7^. Specifically, biomarkers related to lipid biosynthesis and catabolism were associated with OHC exposure in female polar bears, and the associations were more pronounced when sea ice conditions were poor, suggesting that sea ice decline and contaminant exposure have synergistic effects on polar bears^7^. Furthermore, *in vitro* studies indicate that environmental contaminants interfere with adipogenesis in polar bear adipose tissue-derived stem cells as well as with polar bear peroxisome proliferator-activated receptor gamma (PPARG/NR1C3) and pregnane X receptor (PXR/NR1|2)^10,11^, both involved in regulation of energy metabolism^12,13^. Studies on another carnivore, the Baikal seal (*Pusa sibirica*), indicate that polybrominated diphenyl ethers (PBDEs) and several PFASs activate PPAR alfa (PPARA/NR1C1)^14–16^, one of the major regulators of energy metabolism^17^.

Contaminant-mediated adverse effects towards energy metabolism is of particular concern in polar bears due to global climate change. Polar bears use sea ice as a platform to feed on its preferred prey, the ringed seal (*Pusa hispida*)^18^. Decline of the Artic sea ice extent has been related to reduced body condition in polar bears^19–21^, and future reduction in polar bear habitat is suggested to further challenge them energetically^22^. Polar bears go through annual feeding and fasting cycles. When sea ice is present they feed extensively on seals, and during sea ice free phases they go through fasting periods, which may last up to eight months combined with subsequent denning by pregnant females^23,24^. Properly functioning energy metabolism is thus necessary for polar bears to maintain energy supply to vital organs during the periods of food deprivation.

Energy supply during fasting is largely regulated by PPARA/NR1C1. This nuclear receptor protein is highly expressed in tissues with high fatty acid catabolism such as liver, kidney, brown adipose tissue, and heart^25^. PPARA, which forms a heterodimer with retinoic X receptor, is the major regulator of fatty acid catabolism including cellular transport and β-oxidation of fatty acids^17^. High expression of hepatic PPARA during fasting plays a pivotal role in supplying energy to other tissues, as energy released through β-oxidation drives gluconeogenesis from substrates such as glycerol, lactate, and amino acids^26^. PPARA also plays an important role in other metabolic functions such as lipoprotein metabolism and amino acid metabolism^27^. Furthermore, PPARA governs anti-inflammatory processes and is also expressed in immune cells such as peripheral T-cells and macrophages^27–30^.

Transcription of PPARA target genes is enhanced by binding of a ligand, which promotes binding of coactivators and transcriptional activity of target genes^17^. The ligand binding domain (LBD) of PPARA has a large ligand binding pocket (1400 Ångstrøm [Å]), and PPARA is activated by endogenous compounds such as dietary and *de novo* synthetized unsaturated fatty acids and derivatives of arachidonic acid, for example eicosapentaenoic acid, leukotriene B4, 8(S)-hydroxyeicosatetranoic acids and oxidized phospholipids^31–34^. In addition, studies on humans, rodents and seals indicate that some OHCs, such as PFASs, PBDEs and mono[2-ethylhexyl]-phthalate, are PPARA agonists^35–39^, whereas the ability of PCBs and organochlorine pesticides to activate PPARA is largely unknown. Moreover, WY-14643 and other synthetic fibrates, used as dyslipidemic drugs, are also PPARA agonists^34^. However, PPARA-ligand interactions are likely species-specific, because sequences of ligand binding domains (LBD) of nuclear receptors vary between taxa^40^. Understanding species-specific receptor-ligand interactions is particularly important for risk assessment. Although toxic responses of a few test species are often extrapolated to a larger number of species for regulatory purposes, more accurate extrapolation methods have been proposed^41^.

Exposure to environmental contaminants and their potential effects towards energy metabolism is also of high concern in human populations. Exposure to PFASs, PCBs, DDT, bisphenol A and phthalates has been linked to metabolic disorders in animal models and human epidemiological studies^42–45^, but the role of PPARA activation by contaminants for these effects is currently unknown^43^. In comparison to PFAS concentrations found polar bear blood circulation, similar concentrations have been reported in people living in the proximity of one of the world’s largest PFAS manufacturing plants in China, whereas even higher concentrations have been reported for occupationally exposed people^46–49^. Concentrations of PCBs, organochlorine pesticides (except *p,p*’-DDE), and PFAS in polar bear blood circulation on the other hand, generally exceed those reported for humans from northern latitudes^20,50,51^.

The aim of this study was to compare how contaminants and their mixtures that are found at high concentrations in polar bears, or, are of emerging concern for polar bears and humans, affect the transcriptional activity of polar bear vs human PPARA (pbPPARA vs hPPARA). In order to construct a transactivation assay with pbPPARA, we cloned and sequenced pbPPARA from polar bear liver tissue. We examined *in vitro* transactivation of the PPARAs using a luciferase reporter assay. Finally, we compared the exposure concentrations of single compounds to measured or estimated concentrations for polar bear liver.

## Results and Discussion

### Cloning polar bear PPARA and evolutionary conservation

**Figure 1:**
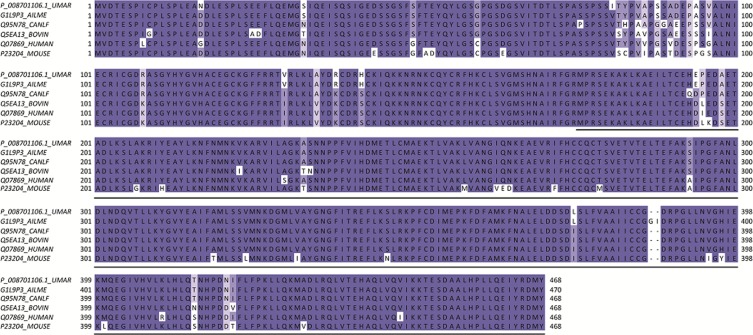
Multiple alignments of the polar bear (UMAR), panda (AILME), dog (CANLF),bovine, human, and mouse PPARA sequence. The following accession numbers were used: polar bear (XP_008701106) from NCBI and panda PPARA (G1L9P3), dog PPARA (Q95N78), bovin (Q5EA13), human PPARA (Q07869), and mouse PPARA (P23204) from UniprotKB^97^. Hinge and ligand binding domains are underlined.

The PPARA hinge and ligand binding domain (LBD) cloned from polar bear liver tissue encoded a protein corresponding to the 117–468 amino acid (AA) region of pbPPARA (Fig. 1). The pbPPARA is very similar to other mammalian PPARAs (Fig. 1). In fact, polar bear PPARA has the same amino acids in 99.4%, 96.6%, 93.8%, 94.2%, and 89.5% of the positions compared to PPARAs from giant panda (*Ailuropoda melanoleuca*), dog (*Canis lupus familiaris*), cattle (*Bos taurus*), human, (*Homo sapiens*) and mouse (*Mus muculus*) (Fig. S1). For the hinge and LBD domain (AA117-468), the identities are slightly higher; 99.7%, 99.0%, 96.6%, 96.6% and 91.1% for giant panda, dog, cattle, human, and mouse respectively (Fig. S1). Cloned pbPPARA sequences of the hinge and LBD was identical to the amino acid sequence predicted from the polar bear genomic sequence (NW_007907208.1).

### Validation of the transactivation assay and cell toxicity

**Figure 2:**
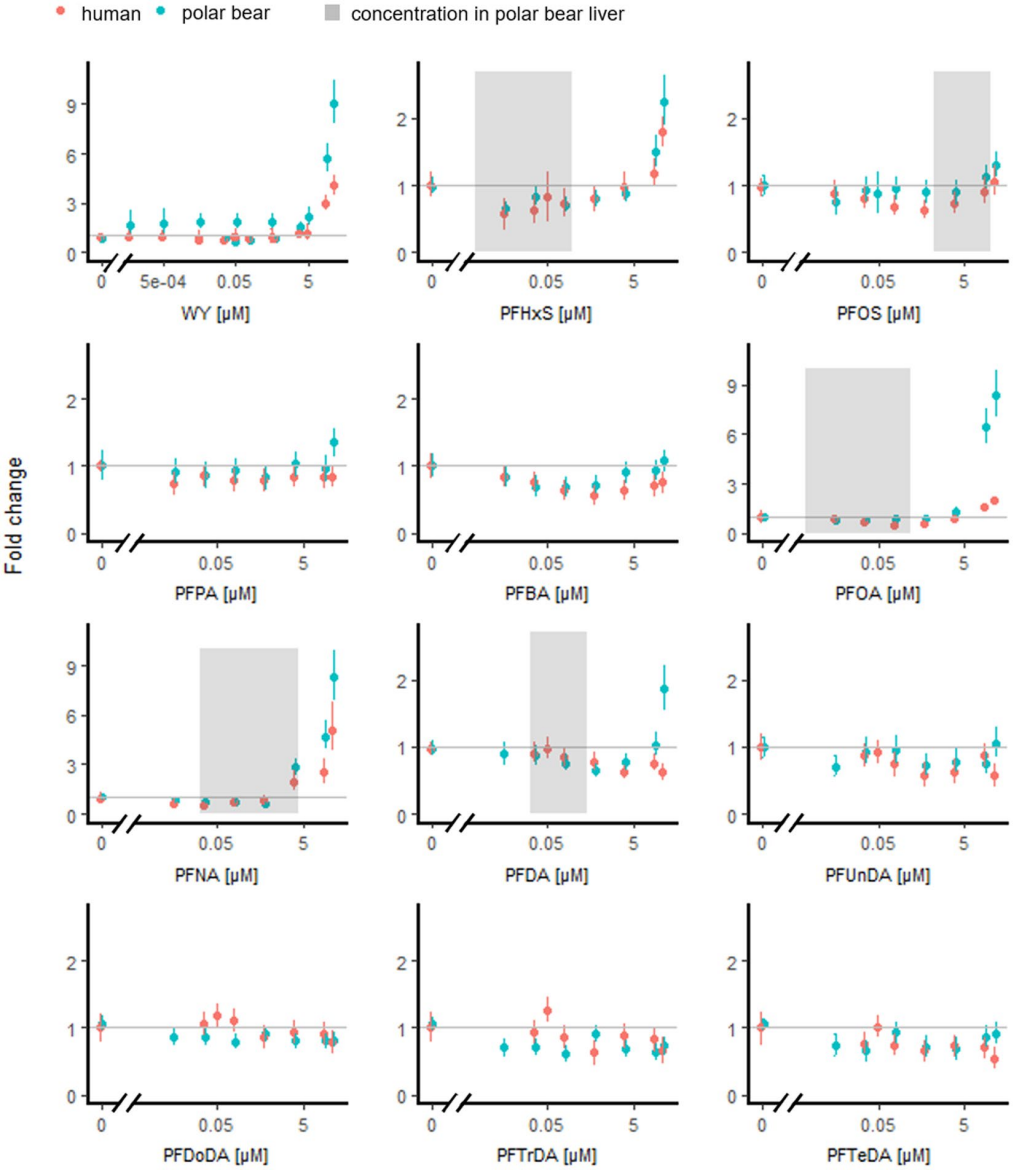
Luciferase activity via human and polar bear PPARA after exposure to WY-14643 and perfluorinated test compounds. Fold changes (mean and 95% confidence intervals) expressed over control, ≤0.5% solvent, are estimated from mixed models with exposure as fixed factor and experiment as random factor. The shaded areas represent estimated or measured liver concentrations in polar bears^47,69,70^ for compounds that affected the fold change >30% at 25 μM (Table 1). Y-axes’ scales vary.

We assume that any luciferase activity we saw in the ligand transactivation assay was due to the activation of PPARA-LBD, and not endogenous nuclear receptors in the cell, as the risk of cross-reactivity by other cellular pathways is highly reduced in the GAL4-construct system^52^. WY-14643 (25 μM) increased pbPPARA-mediated luciferase activity approximately nine-fold in the transiently transfected COS7 cells, whereas hPPARA-mediated luciferase activity was increased four-fold (Fig. 2; Table 1). The PPARA antagonist MK-886 decreased basal luciferase activities mediated by both PPARAs by 55% at 25 μM (Table 1).

**Table 1:** *In vitro* activation of polar bear and human PPARA by known agonist (WY-14643), antagonist (MK-886), and test compounds in GAL4-UAS-based luciferase reporter gene assay performed in COS7 cells.

	Purity (%)	C (μM)	polar bear PPARA		human PPARA	
			Fold Change (95% confidence interval)	Response relative to response by WY (25 μM)	Fold Change (95% confidence interval)	Response relative to response by WY (25 μM)
WY-14643	98	25	9.04 (7.79, 10.49)	100%	4.05 (3.49, 4.7)	100%
MK-886	≥98	25	0.45 (0.33, 0.57)	−7%	0.46 (0.28, 0.64)	−18%
PFHxS	≥98	25	2.25 (1.93, 2.63)	16%	1.8 (1.6, 2.01)	26%
PFOS	≥98	25	1.31 (1.13, 1.49)	4%	1.06 (0.86, 1.31)	2%
PFPA	97	25	1.35 (1.14, 1.56)	4%	0.84 (0.68, 1.01)	−5%
PFBA	98	25	1.08 (0.89, 1.26)	1%	0.77 (0.62, 0.92)	−8%
PFOA	≥95.5	25	8.33 (7.03, 9.85)	91%	2.0 (1.78, 2.21)	33%
PFNA	97	25	8.28 (6.92, 9.91)	91%	5.1 (3.82, 6.82)	135%
PFDA	97	25	1.86 (1.57, 2.21)	11%	0.63 (0.53, 0.75)	−12%
PFUnDA	95	25	1.04 (0.84, 1.29)	1%	0.57 (0.39, 0.75)	−14%
PFDoDA	95	25	0.81 (0.7, 0.93)	−2%	0.78 (0.61, 0.96)	−7%
PFTrDA	97	25	0.74 (0.62, 0.87)	−3%	0.66 (0.48, 0.85)	−11%
PFTeDA	97	25	0.92 (0.76, 1.08)	−1%	0.54 (0.37, 0.7)	−15%
BDE28	>99	25	1.22 (1.08, 1.35)	3%	1.41 (1.3, 1.52)	13%
BDE47	>99	25	1.12 (1, 1.24)	1%	1.38 (1.24, 1.54)	13%
BDE99	>99	25	1.14 (1.01, 1.28)	2%	1.54 (1.35, 1.76)	18%
BDE100	>99	25	1 (0.89, 1.12)	0%	1 (0.89, 1.12)	0%
BDE153	>99	25	1.49 (1.3, 1.68)	6%	1.85 (1.7, 2)	28%
bisphenol A	>99	25	0.83 (0.7, 0.96)	−2%	1.15 (1.04, 1.25)	5%
TBBPA	97	25	0.81 (0.68, 0.94)	−2%	0.92 (0.83, 1.01)	−3%
HBCDD	99	25	0.59 (0.48, 0.7)	−5%	0.82 (0.69, 0.95)	−6%
PCB118	99.2	25	1 (0.86, 1.15)	0%	1.16 (1.01, 1.32)	5%
PCB138	99.99	25	0.6 (0.48, 0.73)	−5%	0.83 (0.71, 0.95)	−6%
PCB153	98.3	25	0.54 (0.42, 0.65)	−6%	0.59 (0.48, 0.69)	−14%
PCB170	99.99	25	0.72 (0.57, 0.88)	−3%	0.83 (0.72, 0.95)	−6%
Aroclor 1254		25	22.2 (18.3, 26.9)	264%	8.76 (6.44, 11.91)	254%
4-OH-CB107	98	15	1.09 (0.97, 1.22)	1%	0.93 (0.76, 1.10)	−2%
4-OH-CB187	98	15	2.2 (2.02, 2.37)	15%	2.27 (1.86, 2.78)	42%
*p,p’*-DDE	≥98	25	0.95 (0.8, 1.1)	−1%	1.49 (1.31, 1.73)	16%
endosulfan	≥98	25	0.71 (0.59, 0.83)	−4%	1.02 (0.9, 1.15)	1%
neutral POP-mixture		6.9	0.81 (0.63, 1)	−2%	0.89 (0.76, 1.02)	−4%
MeSO_2_-POP mixture		0.6	0.7 (0.57, 0.83)	−4%	0.94 (0.82, 1.06)	−2%
OH-POP mixture		0.7	0.14 (0, 0.28)	−11%	0.2 (0.06, 0.33)	−26%

Relative responses, as relative changes in luciferase activity in exposed and solvent-exposed cells, are derived from linear mixed effect models.

**Figure 3:**
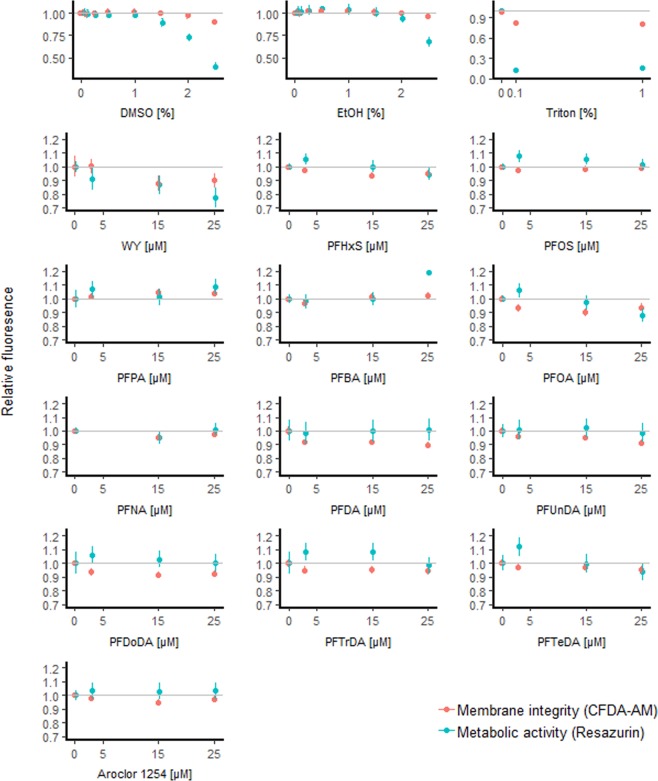
Membrane integrity and metabolic activity in COS7 cells exposed to solvents, Triton X-100, WY-14643, PFASs and Aroclor 1254. Fluorescence (mean and 95% confidence intervals) for solvents, expressed over media, are estimated from linear models. Fluorescence for Triton and test compounds, expressed over ≤1% solvent, are estimated from mixed models with exposure as fixed factor and experiment as random factor. Y-axes’ scales vary.

The viability (β-galactosidase activities, metabolic activity, and membrane integrity) of the COS7 cells was monitored under conditions identical to conditions in the luciferase assay. β-galactosidase activities were relatively constant in cells exposed to different concentrations of all test compounds and mixtures except the synthetic mixture of OH-POPs, which reduced β-galactosidase activities by approximately 50% at the highest concentration. The metabolic activity in COS7 cells was reduced by a maximum of 23% by WY-14643 (25 μM), 6 and 12% by perfluorohexane sulfonate (PFHxS) and perfluorooctanoate (PFOA) (25 μM), and this effect on the metabolic activity was dependent on the concentration of the test compound (Fig. 3; Table S1). In comparison, the positive control Triton X-100 reduced the activity by 84–87% (0.1–1% v/v) (Fig. 3; Table S1). Membrane integrity was reduced by 3–10% by WY-14643, Aroclor 1254, PFHxS, PFOA, perfluoroundecanoate (PFDA), perfluoroundecanoate (PFUnDA), perfluorododecanoate (PFDoDA), perfluorotridecanoate (PFTrDA) and perfluorotetradecanoate (PFTeDA) at 3–25 μM concentrations, but for these compounds the diminished membrane integrity did not seem to be dose dependent (Fig. 3; Table S1). The effects on the membrane integrity were lower, 18–19%, by the positive control Triton X-100 (0.1–1% v/v). The slight decrease in cell viability by WY-14643 and the above-mentioned test compounds may potentially lead to slightly lower luciferase activity in the COS7 cells. The metabolic activity and membrane integrity in COS7 cells was not reduced by perfluorooctane sulfonate (PFOS), perfluorobutanoate (PFBA), perfluoropentanoate (PFPA) or perfluorononanoate (PFNA) (Fig. 3; Table S1). Additionally, the metabolic activity was not reduced by C_10–14_ PFCAs (PFDA, PFUnDA, PFDoDA, PFTrDA or PFTeDA) (Fig. 3; Table S1). Based on previous studies on COS7 cells, hepta- and hexachlorinated PCBs, DDTs or tetrabromobisphenol A (TBBPA) did not lead to concentration-dependent reduction in metabolic activity or membrane integrity^53,54^. Dimethyl sulfoxide (DMSO) or EtOH concentrations at one percent and lower did not affect the viability of the COS7 cells either (Fig. 3).

### *In vitro* activation of polar bear and human PPARA by individual compounds

PFNA and PFOA were strong pbPPARA agonist and they induced reporter gene transcription more than 8-fold (25 μM), similar to the maximal activity induced by the known agonist WY-14643 (Fig. 2; Table 1). PFDA, PFHxS, and PFOS also increased pbPPARA-mediated luciferase activity, but the increase was only 27% or less compared to PFNA and PFOA (Fig. 2; Table 1). PFNA was the most efficient hPPARA agonist with a maximum 5-fold increase in luciferase activity, whereas the luciferase increase by PFOA and PFHxS was ~2-fold (Fig. 2; Table 1). The measured agonistic effects of PFOA, PFNA, PFHxS, and PFOS correspond to previous studies on transactivation of human PPARA in COS-1 and HEK293 cells, mouse PPARA in COS-1 cells, and Baikal seal PPARA in CV-1 cells^15,55,56^. Furthermore, PFDA activated human, mouse, and Baikal seal PPARA in transiently transfected HEK293 cells, COS-1 and CV-1 cells, respectively, but not hPPARA in COS-1 cells^15,55,56^.

C_10–14_ PFCAs reduced basal luciferase activity in cells expressing hPPARA, whereas the basal luciferase in cells expressing pbPPARA was reduced only by C_12-13_ PFCAs (Fig. 2; Table 1). Reduced basal luciferase activity by C_10–14_ PFCAs could be related to reduced viability of COS7 cells; exposure to C_10–14_ PFCAs led to a 5–10% reduction in membrane integrity (Fig. 3). Long-chain PFCAs have previously been shown to have a higher cytotoxic potential in HepG2 cells than medium and short-chain PFCAs^56^. It is unlikely that the observed reduction in basal luciferase activity by long-chain PFCAs is related to antagonistic activity by these compounds, as previous studies suggest that long-chain PFCA are PPARA agonists^15,55,56^.

**Figure 4:**
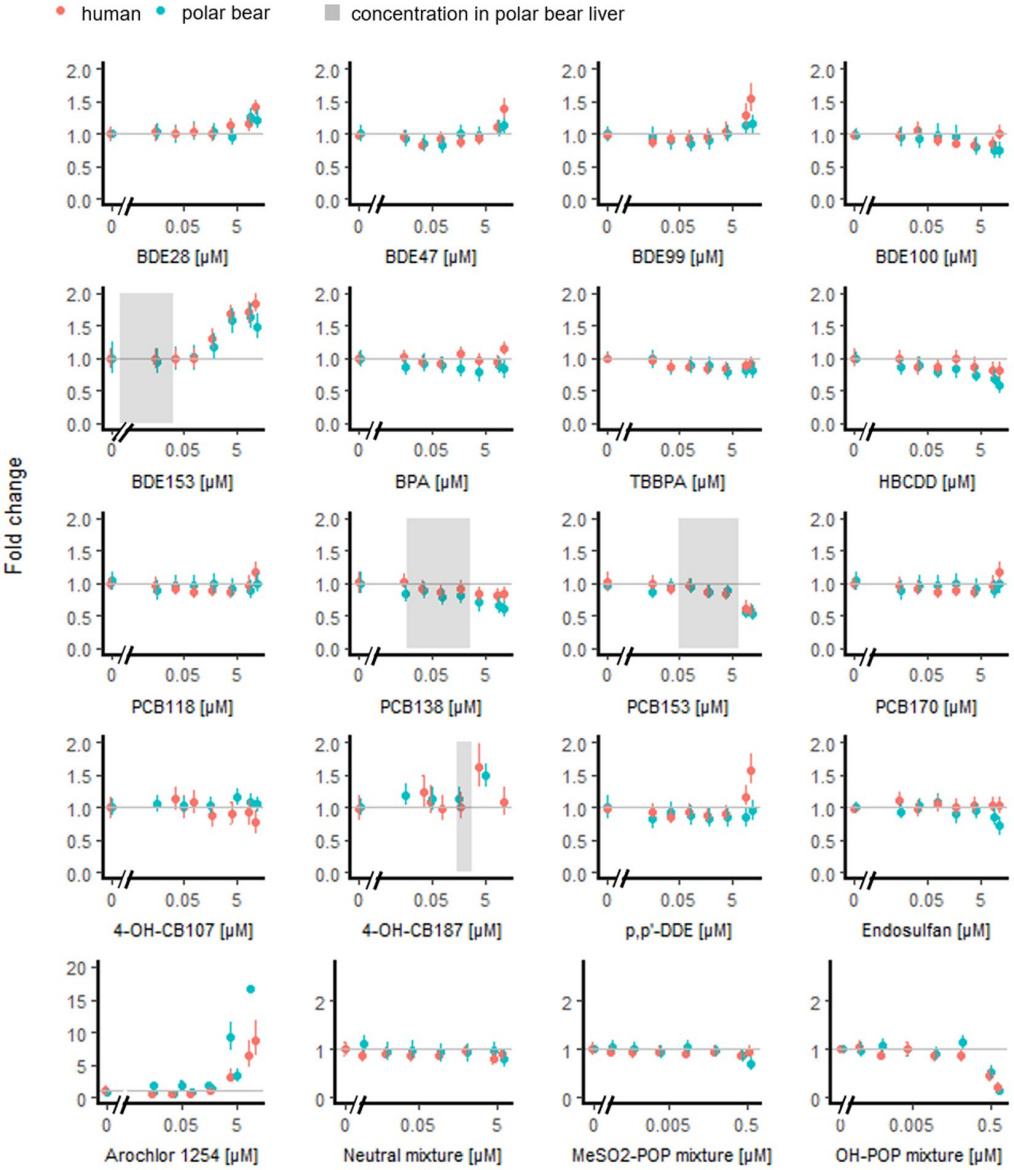
Luciferase activity via human and polar bear PPARA after exposure to brominated and chlorinated test compounds and mixtures. Fold changes (mean and 95% confidence intervals) expressed over control, ≤1% solvent, are estimated from mixed models with exposure as fixed factor and experiment as random factor. The shaded areas represent estimated or measured liver concentrations in polar bears^20,66,70,76^ for compounds that affected the fold change >30% at 25 μM (Table 1). Y-axes’ scales vary.

Polybrominated diphenyl ethers BDE28, BDE47, BDE99, and BDE153 had a weak agonistic effect on polar bear and human PPARA (Fig. 4; Table 1). BDE153 was the most efficient agonist for PPARA among the tested brominated compounds, and induced hPPARA-mediated reporter gene transcription by 1.85 fold at the highest exposure concentration (25 μM). The measured agonistic effects of PBDEs is in agreement with studies on Baikal seal PPARA in CV-1 cells^16^. BDE153 was the most potent activator of Baikal seal PPARA^16^. These results are in contrast with a theoretical structure-activity study suggesting that BDEs would not activate any of the PPAR orthologs^57^. BPA was a weak hPPARA agonist, whereas bisphenol A (BPA) and TBBPA slightly reduced the basal luciferase expression in cells expressing pbPPARA (Fig. 4; Table 1). In a previous study, neither BPA nor TBBPA showed agonistic activity in human cervical carcinoma cells (HGELN cells) stably transfected with GAL4-hPPARA-LBD chimeras^58^.

PCB118 and *p,p’*-DDE were weak hPPARA agonists, whereas PCB138, PCB153, and PCB170 reduced human and polar bear PPARA-mediated basal luciferase activity (Fig. 4; Table 1). To our knowledge, these compounds have not previously been investigated for their ability to activate PPARA. Yet, a study on pb/hPPARG also showed a reduction in basal luciferase activity by PCB153, and further testing confirmed that PCB153 antagonises pb/hPPARG in the presence of a known agonist^11^. 4-OH-CB187 reached the maximum (two fold) PPARA-mediated reporter gene transcription at 15 μM, whereas the transcriptional activities tended to decrease towards the highest concentration (Fig. 4; Table 1).

General activation patterns of human and polar bear PPARA by brominated and chlorinated compounds were similar to those of pb/hPPARG^11^. The transcriptional activity of both receptor constructs was slightly enhanced by the tested brominated compounds, whereas the tested chlorinated compounds showed no effect or reduced basal luciferase activity. This was expected, as the binding pockets PPARA and PPARG are relatively close in size and shape, and they share several common ligands^59,60^. However, the PPARA ligand binding pocket is more lipophilic than that of PPARG^60^.

### *In vitro* activation of polar bear and human PPARA by mixtures

Aroclor 1254 was a strong PPARA agonist, and more than doubled luciferase activity via both polar bear and human PPARA compared to the maximal activity induced by WY-14643 (Fig. 4; Table 1). Aroclor 1254 is a commercially produced PCB mixture composed mainly of mono- and di-*ortho* forms of penta- (~50% of all PCBs) and hexa PCBs (~25% of all PCBs). The strong agonistic effect of Aroclor 1254 could be caused by cocktail effects of different PCBs in the mixture. The synergistic interaction of PCBs and OH-PCBs have been previously shown with the aryl hydrocarbon receptor and estrogen receptor, respectively^61,62^.

None of the synthetic mixtures of neutral POPs, MeSO_2_-POPs, or OH-POPs (Table S2), which were prepared according to the concentrations and composition found in tissues of a 10-year-old male polar bear from Svalbard, Norway^11^, showed agonistic effects towards polar bear or human PPARA (Fig. 4; Table 1). In contrast, basal luciferase activity mediated by pbPPARA was significantly reduced followed by administration of neutral POP and MeSO_2_-POP mixtures. Neutral POP and MeSO_2_-POP mixtures also tended to reduce basal luciferase activity mediated by hPPARA, but not significantly (Fig. 4; Table 1). This may indicate that neutral POPs and MeSO_2_-POPs mixtures have antagonistic properties towards polar bear and human PPARA, as observed for pb/hPPARG^11^. The mixture of neutral POPs consists over 60% of highly chlorinated (≥6 Cl) PCBs (Table S2), and all the highly chlorinated PCBs tested individually (138, 153, 170) reduced human and polar bear PPARA-mediated basal luciferase activity. This leads to a hypothesis that highly chlorinated PCB may have antagonistic properties towards polar bear and human PPARA, which is supported by a study on the commercial PCB mixture Aroclor 1260, mainly composed of hexa- and hepta non-planar PCBs^63,64^ similar to those found in human and polar bear tissues^65,66^. In the study, Aroclor 1260 suppressed hPPARA activation induced by nafenopin in HepG2 cells^65^. The contrasting results between the mixture of neutral POPs and Aroclor 1254 are likely related to different PCB congener composition in the mixtures (Table S2; Fig. S2). The proportion of PCBs (% of molar concentration of ΣPCBs) with 5 or less chlorine atoms was 18 times higher in Aroclor 1254^67^ than in the mixture of neutral POPs (Fig. S2). In addition, Aroclor 1254^67^ contained 92 times more PCBs with a planar configuration having one or less *ortho* chlorine than the mixture of neutral POPs (Fig. S2).

Basal luciferase activity mediated by both polar bear and human PPARA was strongly reduced by the OH-POP mixture (Table 1), which is likely related to the cytotoxicity of the mixture as discussed above.

### Species differences

Fourteen out of 27 test compounds increased transcriptional activity of both polar bear and human PPARA. However, the receptors differed by the type of compounds by which they were transactivated. Polar bear PPARA was both quantitatively and qualitatively more susceptible to transactivation by the tested PFASs than hPPARA (Fig. 2). On the other hand, luciferase activity by PBDEs was slightly greater in cells expressing hPPARA compared to cells expressing pbPPARA, and *p,p’*-DDE and one PCB were also weak hPPARA agonists (Fig. 4). This indicates that transactivation of pbPPAR is initiated by more polar compounds than that of hPPARA. The differences in transactivation patterns between polar bear and human PPARA could possibly be explained by the differences in amino acid sequences in the LBD. Specifically, six substitutions occur between the polar bear and human PPARA LBD; A293S, I375L, R409K, S414T, D419N, and I446V (Fig. 1).

**Figure 5:**
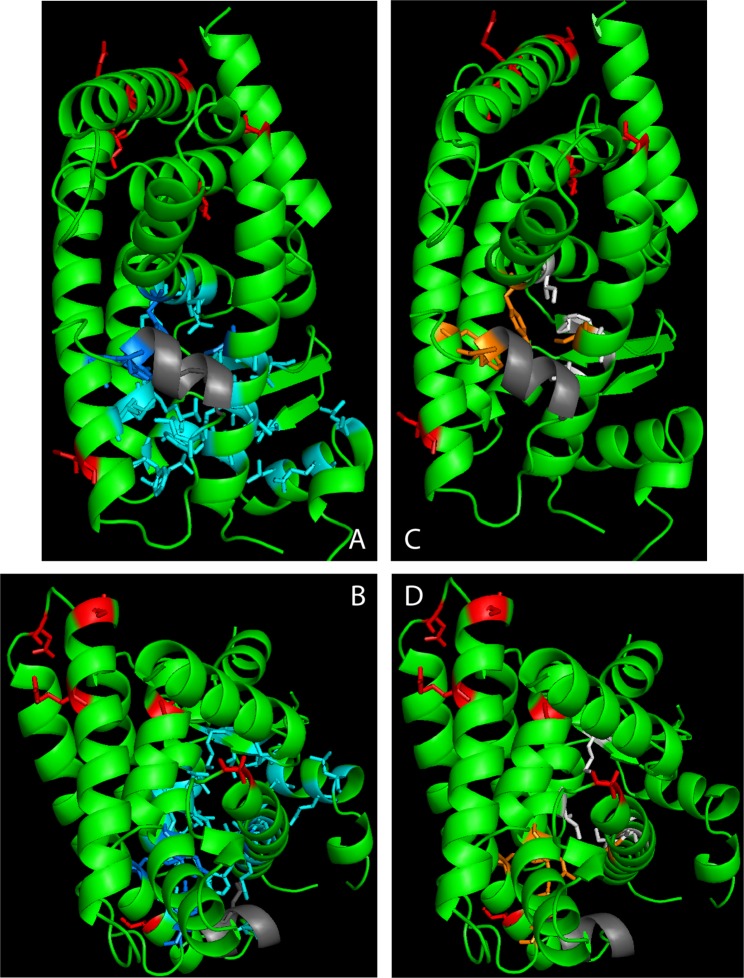
Mapping of substituted amino acids in the human PPARA. The amino acids differing between human and polar bear PPARA were mapped (in red) in a structure of hPPARA (PDB: 3vi8). Helix 12 is shown in grey. To visualize the location of the ligand binding pocket (LBP), amino acids previously shown to participate in the orientation of GW409544 (**A,B**) and WY-14643 (**C**,**D**) were mapped^60,98^. Hydrogen bonding amino acids are shown in dark blue and orange, and amino acids forming hydrophobic interactions are shown in turquoise and white. All substituted amino acids are located away from the LBP.

Mapping of amino acids in a crystal structure of hPPARA (PDB 3VI8) demonstrated that the amino acid positions that hold different amino acids in human and polar bear PPARA are located away from the LBP (>6 Å). Amino acid position 446, however, is localised in helix 11 which contributes to the LBP but appears to be oriented outwards, away from the pocket (Fig. 5). This demonstrates that the substituted amino acids do not participate directly in the orientation of ligands in the PPARA-LBP. Most of these are conservative substitutions with amino acids of similar shape and size, suggesting that they will not significantly alter the binding ability of the receptor. The substitutions in helix 4 (A293S) and the substitution in the turn between helix 10 and 11 (D419N), both lead to introduction of polar amino acids but neither Ser nor Asn is particularly disfavored in helices^68^. Morever, the residues appear to be oriented outward and are unlikely to affect the PPARA structure. It is possible that one or more substitutions may lead to minor changes in the structure of the LBD, which in turn could lead to changes in the binding of ligands, dimerization partners, and cofactors.

### Toxicological significance for polar bears

PFOS followed by PFNA are quantitatively the most abundant PFASs in polar bear liver^69,70^. Activation of pbPPARA by both compounds occurred at environmentally relevant concentrations, whereas PFHxS, PFOA, and PFDA agonised pbPPARA at concentrations which are more than an order of magnitude higher than reported or estimated concentrations in polar bear liver (Fig. 2). The efficacy of PFOS was relatively low, up to 30%, at the concentration range that polar bears are exposed to, whereas PFNA increased pbPPARA-mediated luciferase activity up to almost four-fold at environmentally relevant concentrations (Fig. 4). It is thus highly probable that PFOS and PFNA exposure in free ranging polar bears can activate PPARA and induce transcription of PPARA target genes. As the levels of PFOS and PFNA are very high in some polar bear subpopulations, such as in the Barents Sea subpopulation^9^, PPARA function is likely to be disrupted by these compounds. Converted to liver equivalents, PFOS concentrations range from 1.4 to 21.2 μM^47^, whereas the measured 95% confidence intervals for the other contaminant hotspot subpopulations in the Hudson Bay and East Greenland range from 1.3 to 2.8 μM and 1.03 and 3.22 μM, respectively (confidence intervals for East Greenland subpopulations are estimated from 2 x standard deviation)^69,70^. PFNA concentrations in liver of female polar bears from the Barents Sea are estimated to range from 0.41 to 4.01 μM, whereas the measured concentrations for polar bears from the Hudson Bay and East Greenland subpopulations range from 0.54 to 1.22 μM and 0.02 to 0.73 μM, respectively^47,69,70^. Furthermore, *in vitro* studies on Baikal seal and mouse indicate that PFASs act additively on PPARA at concentrations relevant for polar bears^15,71^. PFNA-mediated changes in PPARA functions are likely to increase in the future, as PFNA concentrations have increased 2.5% per year in Barents Sea polar bears during the period 2000–2014, whereas PFOS concentrations have been stable since 2009^72^. Furthermore, with the declining sea ice the Barents Sea, there is an increase in the use of offshore areas at the eastern Barents Sea by polar bears^73^, and bears using these areas have higher PFAS concentrations than those which stay close to the Svalbard Archipelago^74^.

4-OH-CB187, the most abundant OH-metabolite of PCBs in polar bear tissues^66^, also showed agonistic properties towards pbPPARA at close to environmentally relevant concentrations (Fig. 4). However, it should be noted that the latest measurement of OH-PCBs in polar bear liver is from animals sampled in 1999–2001^66^ and the concentrations have likely increased since then, along with the parent compounds^75^.

BDE153 (and other BDEs) were also weak pbPPARA agonists, but as PBDEs are present at relatively low concentrations in polar bear liver tissue (<0.05 μM^76^; Fig. 4) they are not likely to disturb PPARA activity in free-ranging polar bears.

Transactivation of PPARA by contaminants, in particular PFASs, may have consequences to multiple functions of the body. A correlative field study on over one hundred female polar bears reported contaminant-related alterations in lipid metabolism^7^. Several of the studied biomarkers, such as plasma levels of high density lipoproteins, triglycerides, cholesterol, and lactate, were strongly related to PFAS exposure^7^. Although the authors suggested these relationships may be related to thyroid disruption^7^, the agonistic effects of PFASs on pbPPARA may also contribute to at least the observed relationship between PFASs and high density lipoproteins. As an example, a PPARA agonist in humans raise circulating levels of high density lipoproteins^27,77^. However, it should be kept in mind that polar bear metabolism is highly adapted to cold climate and feeding and fasting cycles^78,79^, and direct comparison of physiological functions between polar bears and humans is thus challenging.

## Methods

### Identification, cloning, and sequencing of polar bear PPARA

We obtained liver tissue from juvenile (2+ year) male polar bear (standard length 185 cm; 124 kg) during the native subsistence hunt in Ittoqqortoormiit/Scoresby Sound, East Greenland (69–71°N, 22–25°W) in March 2012. In collaboration with the local hunters and authorities we were allowed to subsample tissues. The native subsistence hunt and the tissue sampling were carried out in accordance with the Greenland Home Rule (www.lovgivning.gl). CITES import and export permits were obtained to transfer the samples from Greenland to Denmark and Norway. A small piece of liver was placed in RNAlater (AMBION, Austin, TX, USA) within 2 hours after felling. We extracted total RNA from the liver using the TRIzol® Reagent (Invitrogen, Carlsbad, CA, USA) and synthetized cDNA using Superscript II reverse transcriptase and oligo(dT)_20_ primers (both from Invitrogen) following the manufacturer’s instructions. We amplified a partial gene fragment of pbPPARA by gradient PCR using primers designed on the basis of the dog PPARA sequence (NM_001003093) (Table S4) and GoTaq® DNA Polymerase (Promega, Madison, WI, USA). We used SMARTer^TM^ RACE cDNA amplification kit (Clonetech, Mountain View, CA, USA) and gene specific primers (Table S4) to obtain 5′ and 3′-end pbPPARA sequences, but in spite of repeated attempts, a full length 5′-sequence was not obtained (sequence of the first nine amino acids of the A/B domain was not amplified). We confirmed the pbPPARA sequence by sequencing. PPARA-domains were identified based on the similarity to human PPARA (Q07869) from UniProtKB^80^. To compare pbPPARA sequence with closely related mammals we aligned PPARA sequences using Clustal Omega^81^.

### Luciferase reporter assay and exposure to individual compounds

To study transcriptional activity of polar bear and human PPARA by environmental contaminants, we constructed a luciferase reporter assay encoding a fusion protein of the GAL4 DNA-binding domain (DBD; AA1-148) and the hinge and LBD (AA117-468) of pbPPARA or hPPARA. We used similar methods as previously described for a luciferase reporter assay encoding polar bear PPARG^11^. A ligand binding to the LBD enables the Gal4-DBD to bind to its upstream activation sequence. This initiates expression of the luciferase that converts luciferin to oxyluciferin in a chemical reaction emitting measurable light. Briefly, we used COS7 cells, fibrolast-like cells that have been originally derived from the kidney of African green monkey (*Cercopithecus aethiops*)^82^, which are suitable for transfections and express transfected plasmid-DNA^83^. We transiently co-transfected the cells with pCMX-GAL4-pbPPARA or pCMX-GAL4-hPPARA, tk(MH100)x4-luciferase, and pCMV-β-galactosidase at a mass ratio of 1:2:2 using the TransIT®-LT1 Transfection Reagent (Mirus Bio, Madison, WI, USA). Twenty-four hours after transfection we exposed the cells to test chemicals diluted in exposure medium (phenol-free DMEM with 10% stripped FBS) for 24 hours, lysed the cells, and measured enzymatic activity. We tested agonistic effects of the following 27 individual compounds: PFHxS, PFOS, PFBA, PFPA, PFOA, PFNA, PFDA, PFUnDA, PFDoDA, PFTrDA, PFTeDA, BDE28, BDE47, BDE99, BDE100, and BDE153, hexabromocyclododecane (HBCDD), BPA, TBBPA, PCB118, PCB138, PCB153, PCB170, 4-OH-PCB107, 4-OH-PCB187, *p,p’*-DDE and endosulfan. The PBDEs and HBCDD were kindly donated by Åke Bergman, the University of Stockholm. PCB118 was acquired from LGC Standards (Teddington, UK), PCB153 from ChemService (West Chester, PA, USA) and OH-PCBs from Wellington Laboratories Inc. (Guelph, Ontario, Canada). PCB138 and 170 were donated from Krister Halldin (Karolinska Institutet). The remaining compounds were obtained from Sigma-Aldrich (Oslo, Norway). We established the functionality of the luciferase assay by exposure to the known PPARA agonist WY-14643 (Sigma Aldrich) and antagonist MK-886 (Tocris Biosciences)^32,84^. Purities of all ligands are listed in Table 1^85–87^. Exposure concentrations were environmentally relevant and ranged from 0.5 pM–25 μM. OH-PCBs, PFDoDA, PFTrDA and PFTeDA were dissolved in EtOH, whereas the remaining compounds were dissolved in DMSO (grade: Hybri-max; Sigma-Aldrich). The solvent percentages were kept at 0.5% for PFASs and 1% for brominated and chlorinated compounds. Each exposure of individual compounds was performed in triplicates and repeated at least three times.

### Exposure to mixtures of POPs

We tested agonistic effects of the following synthetic mixtures on polar bear and human and PPARA: the mixture of neutral POPs, the mixture of MeSO_2_-metabolites of POPs, the mixture of (OH) POPs, and a commercial mixture Aroclor 1254, all dissolved in pure DMSO. The three synthetic mixtures of POPs were prepared according to the concentrations and composition found in tissues of a 10-year-old male polar bear from Svalbard, Norway, as described previously^11^. Briefly, the mixtures included (1) 44 neutral POPs, (2) 16 MeSO_2_-metabolites of POPs and (3) seven OH-POPs (Tables S2 and S3). The concentrations of synthetic mixtures of neutral POPs and MeSO_2_-POPs, 6.9 and 0.59 μM, respectively, reflected levels measured in polar bear adipose tissue, which are slightly higher compared to concentrations found in polar bear liver^88^. The highest concentration of the OH-POP mixture, 0.74 μM, was composed based on estimated liver concentrations multiplied by six of the measured concentrations in adipose tissue^88^. However, concentrations of OH-POPs in the adipose tissue are likely underestimated due to low recovery of OH-PCBs extracted using a semipermeable nonpolar membrane. Exposures of Aroclor 1254 were performed in triplicates and repeated five to seven times. Exposures of neutral POPs, MeSO_2_-POPs and OH-POPs were performed in triplicates and repeated twice. The solvent concentration was kept at 1% for the synthetic mixtures.

### COS7 viability

We monitored the general viability of COS7 cells through stability of β-galactosidase activities. In addition, we tested the effect of PFASs and Aroclor 1254 up to 25 μM on the metabolic activity and the membrane integrity of COS7 cells using a resazurin (Sigma Aldrich) reduction assay and fluorogenic dye 5-carboxyfluorescein diacetate acetoxymethyl ester (CFDA-AM, Fisher Scientific)^89,90^, whereas the effect of hepta- and hexachlorinated PCBs, DDTs and TBBPA on COS7 cell viability have been tested as a part of other studies^53,54^. Triton X-100 at 0.1 and 1% (v/v) was used as positive controls for the cell viability assays. Each measurement was performed in triplicates and repeated three times. Furthermore, we tested the effect of solvent concentrations (up to 2.5%) on the metabolic activity and the membrane integrity of COS7 cells once in triplicates^89,90^.

### Data analyses

We used the statistical program R version 3.3.1. for data analyses^91^. Luciferase activities, measured as luminescence (RLU), were corrected for differences in transfection efficiencies by normalized β-galactosidase activities (A_420 nm_). Responses were expressed as the ratio of normalized luciferase activities in cells exposed to test compounds over cells exposed to solvent (fold). We used linear mixed effect models to investigate effects of contaminants and their mixtures on transactivation of polar bear and human PPARA, and on the viability of COS7 cells. The models were run separately for each compound/mixture. Exposure concentration was used as a categorical fixed factor and the experiment as a random factor. The significance level was set to α = 0.05. We used diagnostic plots of residuals to verify that the model assumptions were met; most importantly to show constant variance between residuals^92^. pbPPARA mediated responses by WY-14643, Aroclor 1254, BDE100, PFHxS, PFOA, PFNA, PFDA, and PFUnDA, as well as hPPARA mediated responses to WY-14643, Aroclor 1254, PFOS, PFNA, PFDA, BDE47, BDE99, BDE100, PCB118, and *p,p’*-DDE exposure were ln-transformed for the mixed models to better approximate model assumptions. Omittance of one to two outliers from models explaining pbPPARA mediated responses to PFBA, or hPPARA-mediated responses to 4-OH-PCB107, BDE28, and TBBPA, did not change the significance of the reported results. We attempted to fit mixed effect dose-response curves to the data using a four parameter log-logistic model with experiment as a random factor in R library *medrc*^93^, but the data coverage was insufficient for almost all compounds. The curves were not thus fitted for any of the compounds, and concentrations corresponding to the midway between the estimates of the lower and upper plateaus (EC50) were not estimated.

To assess if our results were environmentally relevant, we compared our exposure concentrations to contaminant concentrations in polar bear liver^66,69,70^. Additionally, as PFASs are not routinely measured in liver of polar bears from Svalbard, we estimated liver concentrations for this population based on concentrations reported for plasma^47^ as reported elsewhere^9^. We converted PFAS concentrations from plasma to whole blood to liver using PFAS mass fractions determined for human plasma^94^, (haematocrit for polar bears is similar to humans)^95^ and PFAS concentration ratios of whole blood/liver in polar bears^96^. To estimate liver concentrations of lipophilic compounds in polar bears from Svalbard to Alaska, we used contaminant data from fat tissue^20,70,76^, and converted it to liver concentrations based on tissue distribution of these compounds in polar bears^66^.

All data are made available on the Norwegian Polar Institute data repository (data.npolar.no).

## Supplementary information

Supplementary information
