# Supplementary material for: Environmental contaminants modulate the transcriptional activity of polar bear (Ursus maritimus) and human peroxisome proliferator-activated receptor alpha (PPARA)
Source: Sci Rep. 2019 May 6;9:6918. doi: 10.1038/s41598-019-43337-w (PMC6502799; doi:10.1038/s41598-019-43337-w)
Supplement: Supplementary file 1 — Supplementary information [file 41598_2019_43337_MOESM1_ESM.pdf]

Supporting information for:

Environmental pollutants modulate the transcriptional activity of polar bear (*Ursus maritimus*) and human peroxisome proliferator-activated receptor alpha (PPARA)

*Heli Routti *,[1], Mari K. Berg [1,2], Roger Lille-Langøy [2], Lene Øygarden [1,2], Mikael Harju [3], Rune Dietz [4], Christian Sonne [4], Anders Goksøyr [2]*

[1] Norwegian Polar Institute, Fram Centre, NO-9296 Tromsø, Norway

[2] Department of Biological Sciences, University of Bergen, NO-5020 Bergen, Norway

[3] Norwegian Institute for Air Research, Fram Centre, NO-9296 Tromsø, Norway

[4] Aarhus University, Department of Bioscience, Arctic Research Centre, DK-4000, Roskilde, Denmark

Table S1. Metabolic activity (resazurin reduction) and membrane integrity (CDFA-AM) in COS7 cells exposed to Triton X-100 (positive control), WY-14643, Aroclor 1254, and PFASs. Mean fluorescence (with 95% confidence intervals) expressed as percentage over solvent control (⩽1%), are predicted from mixed models with exposure as fixed factor and experiment as random factor.

Compound	C (μM)	Metabolic activity (% over solvent)	Membrane integrity (% over solvent)
Triton X-100	0.01	13% (9, 16)	82% (80, 85)
Triton X-100	0.001	16% (13, 19)	81% (79, 82)
WY-14643	25	77% (70, 84)	90% (84, 95)
Aroclor 1254	25	103% (97, 109)	97% (95, 99)
PFHxS	25	94% (90, 99)	95% (92, 97)
PFOS	25	101% (97, 105)	99% (97, 101)
PFBA	25	119% (114, 124)	102% (100, 105)
PFPA	25	109% (103, 115)	104% (102, 106)
PFOA	25	88% (83, 93)	94% (91, 96)
PFNA	25	101% (96, 106)	97% (95, 100)
PFDA	25	100% (93, 108)	90% (88, 92)
PFUnDA	25	98% (92, 105)	91% (89, 93)
PFDoDA	25	100% (94, 106)	92% (90, 95)
PFTrDA	25	98% (92, 105)	94% (92, 97)
PFTeDA	25	94% (87, 100)	95% (92, 98)

Table S2. Concentrations (nM) of POPs in the synthetic mixture of neutral POPs and Aroclor 1254.[1]

	n(Cl)	n(*ortho*-Cl)	Mixture of neutral POPs (nM)	Aroclor 1254 (nM)
PCB 18	3	2	2.4	
PCB 28+31	3	1	3.4	
PCB 33	3	1		
PCB 40	4	2		74.7
PCB 41+64	4	2		266.7
PCB 47+48	4	2		81.4
PCB 49	4	2		347.5
PCB 52	4	2	7.2	584.7
PCB 66+	4	1		2278.8
95	5	3		
PCB 70	4	1		1213.6
PCB 74	4	1		380.3
PCB 77	4	0		371.8
PCB 81	4	0		4.0
PCB 82+	5	2		525.6
151	6	3		
PCB 83	5	2		105.0
PCB 85	5	2		87.5
PCB 87+	5	2		1093.1
115	5	2		
PCB90+	5	2		1636.4
101	5	2	79.9	
PCB 91	5	3		522.9
PCB 92	5	2		786.4
PCB 97	5	2		248.5
PCB 99	5	2	137.9	758.7
PCB 105	5	1	0.8	2212.4
PCB 110	5	2		2001.2
PCB 114+122	5	1		10.1
PCB 118	5	1	12.9	3068.0
PCB 123	5	1		33.1
PCB 126	5	0		41.7
PCB 128	6	2	9.7	184.8
PCB 129	6	2		199.5
PCB 132	6	3		650.7
PCB 135	6	3		210.0
PCB 137+	6	2		46.2
176	7	4		
PCB 138	6	2	113.6	1442.8
PCB 141+	6	2	59.8	276.8
179	7	4		
PCB 146	6	2		207.1
PCB 149	6	3	191.2	620.6
PCB 153	6	2	1136.1	726.7
PCB 156	6	1	74.8	616.9
PCB 157	6	1	4.1	294.8
PCB 167	6	1	3.6	
PCB 169	6	0		0.3
PCB 170	7	2	657.7	74.8
PCB 173	7	3		25.3
PCB 174	7	3		499.0
PCB 177	7	3		24.9
PCB 180	7	2	1037.1	97.8
PCB 183	7	3	48.9	33.6
PCB 187	7	3	102.1	39.4
PCB 189	7	1	2.2	
PCB 194	8	2	826.0	
Sum PCBs			4512	25006

	Mixture of neutral POPs (nM)	Aroclor 1254 (nM)
Hexachlorobenzene	49.41	
α-hexachlorocyclohexane	2.26	
β-hexachlorocyclohexane	49.79	
Heptachlor epoxide	330.65	
Oxychlordane	1833.08	
cis-chlordane	1.21	
trans-nonachlor	13.1	
cis-nonachlor	0.25	
Mirex	30.11	
p.p'-DDE	36.48	
p.p'-DDD	1.45	
p.p'-DDT	2.24	
PBDE 17	0.01	
PBDE 28	0.05	
PBDE 47	15.31	
PBDE 66	0.09	
PBDE 100	2.42	
PBDE 99	12.46	
PBDE 85	0.57	
PBDE 154	0.78	
PBDE 153	1.22	
PBDE 138	0.13	
Sum all POPs	6895	25006

Table S3. Composition of synthetic mixtures of a) MeSO2-POPs and b) OH-POPs.

Compound	nM
a)	
3-$MeSO_2$-PCB49	41.73
4-$MeSO_2$-PCB49	42.57
3-$MeSO2_2$-PCB91	0.00
4-$MeSO_2$-PCB91	19.51
3-$MeSO_2$-PCB101	141.85
4-$MeSO_2$-PCB101	129.58
3-$MeSO_2$-DDE	1.92
3-$MeSO_2$-PCB87	77.74
3-$MeSO_2$-PCB110	0.00
4-$MeSO_2$-PCB110	18.64
3-$MeSO_2$-PCB149	6.15
4-$MeSO_2$-PCB149	47.10
3-$MeSO_2$-PCB132	0.41
4-$MeSO_2$-PCB132	20.89
3-$MeSO_2$-PCB141	18.65
4-$MeSO_2$-PCB141	16.10
3-$MeSO_2$-PCB174	1.25
4-$MeSO_2$-PCB174	2.18
SUM	586.27

b)	
Pentachlorophenol	45.05
4-OH-HpCS	8.11
4-OH-PCB 107	150.41
4-OH-PCB 146	56.21
4-OH-PCB 187	274.70
4-OH-PCB 172	70.72
4'-OH-PCB 193	136.51
SUM	741.72

Table S4. Primer sequences used for cloning and identification of polar bear PPARA from liver tissue.

Primer	Sequence (5'-3')	Area of use
MT84	TTCCGGCGAACCATCCGGC	Amplification of initial pbPPARA segment, fwd
MT85	GCTGCGTCCGACTCCGTCTT	Amplification of initial pbPPARA segment, rev
MT613	GTTTCTTGAATTCATGCCAAGATCGGAAAAAGCG	Amplification of pbPPARA-LBD, fwd; EcoRI
MT614	GTTTCTTGGATCCTCAGTACATGTCCCTGTAG	Amplification of pbPPARA-LBD, rev; BamHI
MT631	GTTTCTTGAATTCATGCCAAGATCTGAGAAAGC	Amplification of hPPARA-LBD, fwd; EcoRI
MT632	GTTTCTTGGATCCTCAGTACATGTCCCTGTAG	Amplification of hPPARA-LBD, rev; BamHI
MT529	GCACGTGCTCAAACTTCACTTGCAGACC	3´- RACE GSP, pbPPARA
MT600	AGCTTGTGCAGGTCATCAAGAAGACGG	3´- RACE NGSP, pbPPARA

a)

Ortholog		Polar bear PPARA	Panda PPARA	Dog PPARA	Bovine PPARA	Human PPARA	Mouse PPARA	
		117-468	117-470	117-468	117-468	117-468	117-468	Amino acids
Polar bear PPARA	1-468		99.7%	99.0%	96.6%	96.6%	91.1%	Hinge and ligand binding domain
Panda PPARA	1-470	99.4%		98.6%	96.3%	96.3%	90.8%	
Dog PPARA	1-468	96.6%	96.8%		96.9%	96.9%	91.5%	
Bovine PPARA	1-468	93.8%	94.0%	95.3%		96.6%	92.5%	
Human PPARA	1-468	94.2%	94.2%	94.7%	94.9%		92.2%	
Mouse PPARA	1-468	89.5%	89.8%	90.2%	91.2%	92.3%		
	Amino Acids	Full-length						

b)

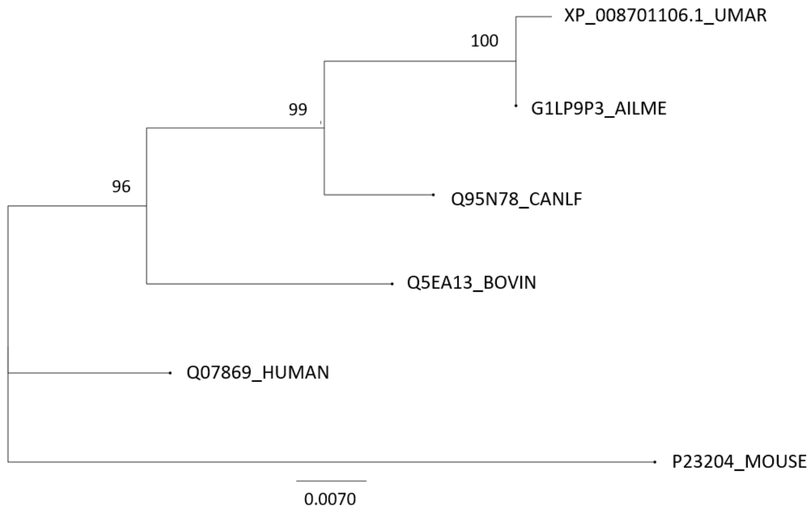

Figure S1. Degree of similarity (a) and a phylogenetic tree (b) for polar bear, panda, dog, bovine, human, and mouse PPARA sequence. The following accession numbers were used: polar bear (XP_008701106) from NCBI, and panda PPARA (G1L9P3), dog PPARA (Q95N78), bovin (Q5EA13), human PPARA (Q07869), and mouse PPARA (P23204) from UniprotKB.[2]

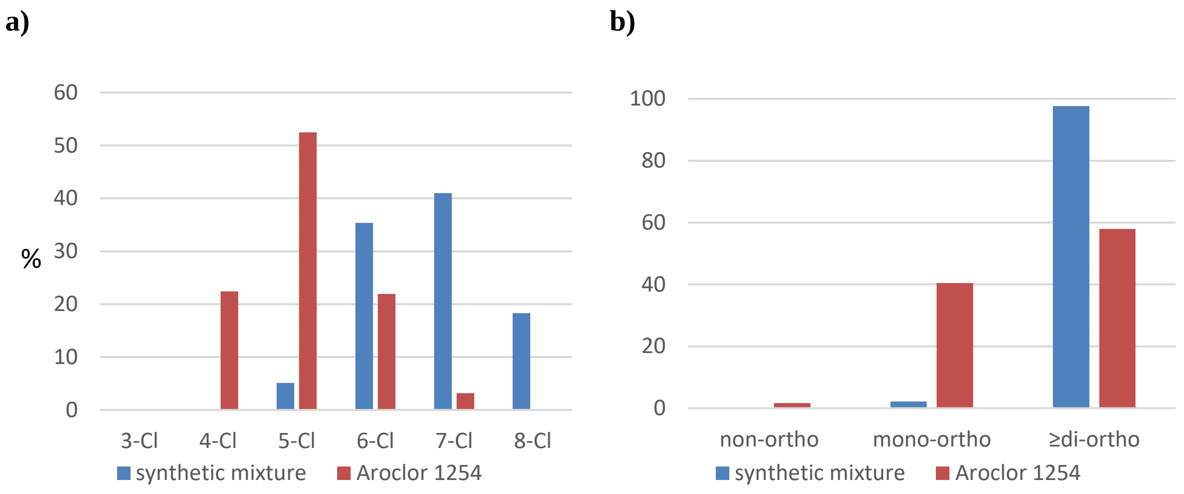

Figure S2. PCB congeners (% of molar concentration) based on a) the number of chlorine (Cl) atoms and b) the number of *ortho*-substitutions in synthetic mixtures and Aroclor 1254.[1]

References:

1 Kodavanti, P. R. S. *et al.* Differential effects of two lots of Aroclor 1254: Congener-specific analysis and neurochemical end points. *Environ. Health Perspect.* **109**, 1153-1161, doi:10.1289/ehp.011091153 (2001).

2 The UniProt, Consortium. Update on activities at the Universal Protein Resource (UniProt) in 2013. *Nucleic Acids Res.* **41**, D43-D47, doi:10.1093/nar/gks1068 (2013).
